# Unmasking a two-faced protein

**DOI:** 10.7554/eLife.83482

**Published:** 2022-10-19

**Authors:** Ivan Maslov, Jelle Hendrix

**Affiliations:** 1 Dynamic Bioimaging Lab, Advanced Optical Microscopy Centre, Biomedical Research Institute, Hasselt University Hasselt Belgium

**Keywords:** protein dynamics, single-molecule FRET, single molecule fluorescence, postsynaptic density, *E. coli*

## Abstract

Single-molecule fluorescence spectroscopy and molecular dynamics simulations illuminate the structure and dynamics of PSD-95, a protein involved in neural plasticity.

**Related research article** Hamilton GL, Saikia N, Basak S, Welcome FS, Wu F, Kubiak J, Zhang C, Hao Y, Seidel CAM, Ding F, Sanabria H, Bowen ME. 2022. Fuzzy supertertiary interactions within PSD-95 enable ligand binding. *eLife* **11**:e77242. doi: 10.7554/eLife.77242.

To help you process this article, billions of neurons in your brain have been learning for years how to convert images and letters on the screen into words and ideas. The biochemical background for learning and memory is neuroplasticity: neurons emerge and die, form new synapses, and abandon old ones. The strength of each connection is fine-tuned by protein molecules communicating with each other inside a synapse.

One of the most abundant proteins in the postsynaptic density – the area of the neuron where nerve signals are amplified or repressed – is a protein called postsynaptic density 95 (PSD-95). This protein modulates interactions between hundreds of other proteins (Figure 1A; Wang et al., 2010; Fernández et al., 2009), and its structure and function change depending on synaptic activity (Bissen et al., 2019). Could it be that the different poses, or conformations, of the domains within PSD-95 specify which partners this protein interacts with inside the neuron, and hence allow neuroplasticity?

**Figure 1.**
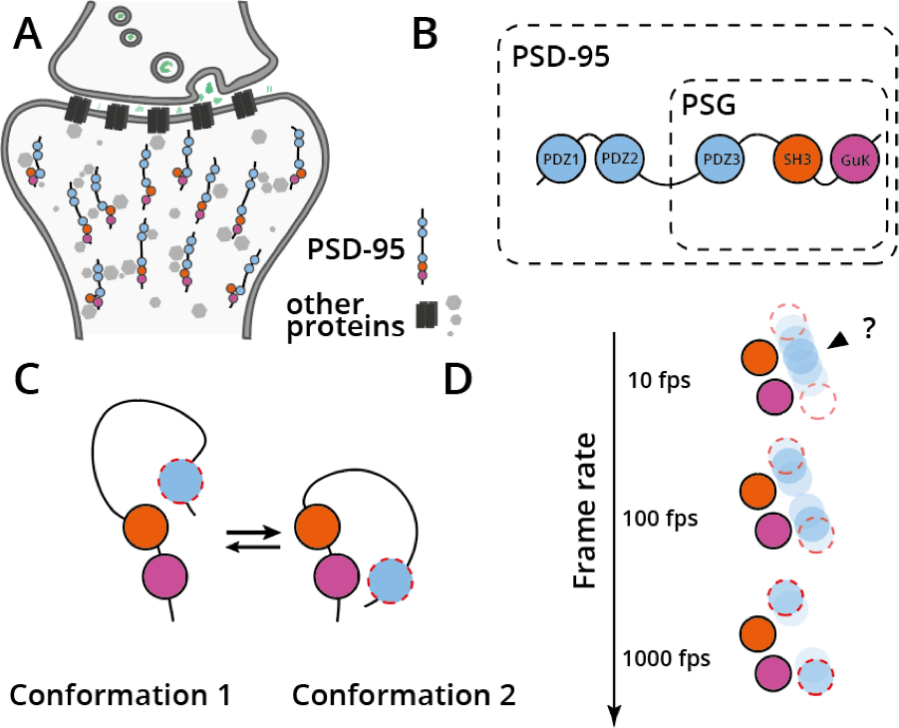
Synaptic localization, structure and dynamics of protein PSD-95. (**A**) Protein PSD-95 (shown as five circles of different colors on a line) mediates protein-protein interactions in the postsynaptic density (other proteins are shown as grey circles or black squares) and hence modulates signal transduction in neurons (neurotransmitters are shown in green being released from the pre-synaptic cell). (**B**) Domain structure of PSD-95, showing the five domains of the protein (with the PDZ domains shown in light blue, the SH3 domain shown in orange and the GuK domain shown in pink). The PSG supramodule is indicated. (**C**) The PSG supramodule switches quickly between two conformations in which the PDZ3 domain (shown in light blue with a dashed red border) takes different positions. (**D**) High-speed recording of structural data overcomes averaging of distinct protein conformations. Top: when observed at 10 frames per second (fps), the positions of the SH3 and GuK domains (which remain still) are correctly resolved, but the PDZ3 domain is switching between its positions in the two conformations of the PSG supramodule faster than the images are being taken. This results in an ‘average’ conformation being recorded (shown in blue) instead of the two ‘real’ conformations with the correct positions of the PDZ3 domain (red dashed circles). Center: when observed at 100 fps, a similar thing happens, but in this case two (incorrect) averaged conformations are observed. Bottom: 1000 fps is a high enough frame rate to correctly resolve the two conformations of the PSG supramodule. Observed averaged conformations are shown in blue and marked with an arrowhead; true underlying conformations are shown as red dashed circles.

Now, in eLife, a group led by Mark Bowen (from Stony Brook University), Feng Ding and Hugo Sanabria (both from Clemson University) – with George Hamilton, Nabanita Saikia and Sujit Basak as joint first authors – reports on a structural model of PSD-95 based on single-molecule fluorescence microscopy and computer simulations. The group also confirms the predictions of the proposed model with a complimentary biochemical technique called disulfide screening, and reveal how ligand binding by one protein domain within PSD-95 is assisted by the proximity of another domain (Hamilton et al., 2022).

PSD-95 consists of five domains: three PDZ domains (PDZ1, PDZ2, PDZ3), an SH3 domain, and a GuK domain (Figure 1B). Three of these domains – PDZ3, SH3, and GuK – associate closely with each other and form a conserved supramodule called PSG. The two other domains, PDZ1 and PDZ2, are separated from PSG by a long flexible linker, and mostly interact with each other rather than with the other domains (McCann et al., 2012; McCann et al., 2011). Within PSG, the interaction between domains SH3 and GuK is tight, while the PDZ3 domain can ‘wiggle’ around them (Korkin et al., 2006; Tavares et al., 2001). In computer simulations, the PDZ3 domain can reach both its closest neighbor, the SH3 domain, and the more distant GuK domain, but it remained unclear whether both or only one of the two PDZ3 conformations occur in nature (Figure 1C; Korkin et al., 2006).

Hamilton et al. used single-molecule fluorescence microscopy to track the conformational dynamics of PSG within full-length PSD-95, showing that the PDZ3 domain switches between two major conformations with respect to the SH3-GuK complex (Figure 1C). In particular, they used a technique called single-molecule Förster resonance energy transfer (smFRET) – frequently referred to as a ‘molecular ruler’ – to measure the distances between the domains in PSG. Combining these measurements with a simulation technique called ‘rigid-body docking’ revealed the atomic structures of both conformations.

The conformations observed via smFRET are in good agreement with those that Hamilton et al. describe using discrete molecular dynamics simulations. In one conformation, the PDZ3 domain is close to the SH3 domain without contacting the GuK domain, while in the other, the PDZ3 domain is bound to the GuK domain. To validate the proposed atomic structures Hamilton et al. used disulfide screening. This technique relies on the fact that cysteine residues will form disulfide bonds with each other quicker when they are in close proximity. By substituting amino acid residues in PSD-95 for cysteines, Hamilton et al. were able to determine that residues that were presumed to be close together based on the proposed structures formed disulfide bonds faster than randomly selected pairs of residues.

Often, in realtime structural investigations like the ones performed by Hamilton et al., if a protein switches between two conformations faster than the detector can capture, the observed conformation will be a ‘blurred’ average, equally distant from the two extreme protein states (Figure 1D). Previous experiments performed on the PSG supramodule used smFRET to capture slow protein dynamics, and were only able to capture a single structure for the module (McCann et al., 2012). This result was inconsistent with structures for PSD-95 determined using other biophysical methods, such as SAXS or NMR (Zhang et al., 2013).

The observation by Hamilton et al. that PSD-95 fast-switches between its two conformations solves this almost decade-old contradiction: experiments that only capture slow dynamics result in an ‘averaged’ structure of the protein, while capturing the fast dynamics of PSD-95 reveals the two extreme conformations that lead to that average. Hamilton et al. captured both fast and slow protein dynamics using two types of single-molecule FRET microscopes, and their structural model containing two states for PSG now agrees nicely with data from other techniques.

Finally, Hamilton et al. demonstrated that interactions between protein domains in the PSG supramodule are critical for the activity of PSD-95, and, in particular, for its interaction with neuroligin. Negative electric charges from the SH3 domain balance positive charges near the ligand binding pocket of the PDZ3 domain to allow PSD-95 to bind to the positively charged ligand. Additionally, Hamilton et al. showed that structural dynamics of PSG are fine-tuned by the two other domains (PDZ1 and PDZ2). This tuning results in complex local changes of structural dynamics on the timescales from microseconds to seconds.

The techniques used by Hamilton et al. pave the way for future insights into natural interactions between domains in the same protein. This may facilitate the design of more selective drugs targeting inter-domain protein interfaces, as well as de novo design of multi-domain proteins for biotechnological applications. Furthermore, the work of Hamilton et al. builds a solid foundation for further intriguing investigations. For example, how are the conformations and structural dynamics of PSD-95 affected by ligands, protein partners, or post-translational modifications? It will also be important to determine whether there are protein partners and ligands that preferentially bind PSD-95 in the conformation in which the PDZ3 domain directly interacts with the Guk domain? This is, definitely, something for us and the neurons in our brains to learn in the future.
